# Facing Facts: Facial Injuries from Stand-up Electric Scooters

**DOI:** 10.7759/cureus.6663

**Published:** 2020-01-15

**Authors:** Mohamedkazim Alwani, Alexander J Jones, Morgan Sandelski, Elhaam Bandali, Benjamin Lancaster, Michael W Sim, Taha Shipchandler, Jonathan Ting

**Affiliations:** 1 Otolaryngology, Indiana University School of Medicine, Indianapolis, USA; 2 Otolaryngology - Head and Neck Surgery, Indiana University School of Medicine, Indianapolis, USA; 3 Public Health, Richard M. Fairbanks School of Public Health, Indianapolis, USA

**Keywords:** stand-up scooters, bird, lime, trauma

## Abstract

Background

Stand-up electric scooters (SES) are a popular public transportation method. Numerous safety concerns have arisen since their recent introduction.

Methods

A retrospective chart review was performed to identify patients presenting to the emergency departments in Indianapolis, who sustained SES-related injuries.

Results

A total of 89 patients were included in our study. The average patient age was 29 ± 12.9 years in a predominantly male cohort (65.2%). No patient was documented as wearing a helmet during the event of injury. Alcohol intoxication was noted in 14.6% of accidents. Falling constituted the leading trauma mechanism (46.1%). Injuries were most common on Saturday (24.7%) from 14h00 to 21h59 (55.1%). Injury types included: abrasions/contusions (33.7%), fractures (31.5%), lacerations (27.0%), or joint injuries (18.0%). The head and neck region (H&N) was the most frequently affected site (42.7%). Operative management under general anesthesia was necessary for 13.5% of injuries. Nonoperative management primarily included conservative orthopedic care (34.8%), pain management with nonsteroidal anti-inflammatory drugs (NSAIDs) (34.8%) and/or opioids (4.5%), bedside laceration repairs (27.0%), and wound dressing (10.1%). Individuals sustaining head and neck injuries were more likely to be older (33.8 vs. 25.7 years, p=0.003), intoxicated by alcohol (29.0% vs. 3.9%, p=0.002), and requiring CT imaging (60.5% vs. 9.8%, p <0.001).

Conclusion

Although SESs provide a convenient transportation modality, unregulated use raises significant safety concerns. More data need to be collected to guide future safety regulations.

## Introduction

The Standup Electric Scooter (SES) is a recent implementation in the public transportation system. It was introduced in September 2017 by an electric vehicle sharing company, Bird® Rides, Inc. in Santa Monica, California [1]. Other prominent companies, such as Lime® (Washington, D.C.) and Spin® (California), have also emerged on the market [2-4]. These companies supply a dockless, rentable, transportation-sharing system similar to that of bike-sharing systems. SESs have provided an environmentally friendly alternative to gasoline-powered vehicles with the goal of decreasing traffic congestion and carbon emissions for short-distance transportation [2-4]. Now featured in hundreds of US cities and college campuses, SESs have gained immense popularity for their convenience, availability, and affordability [2-4]. The scooters are found and left at arbitrary locations throughout designated service areas, and riders are charged per minute of use. The scooters can be utilized by anyone with a valid driver’s license, credit card, and a smartphone application [1-4]. The scooters can reach speeds just under 15 mph (24.1 kph) with a range of approximately 20 miles (32.2 km) [1-2].

Although SESs may provide some environmental and traffic benefits, their increasing popularity in larger cities, combined with vague regulations for use, raises a concern about potentially negative public health implications. While the terms of use defer to local laws and regulations, companies strongly recommend operators to avoid sidewalks, adhere to traffic safety laws, and always wear a helmet [2-4]. Furthermore, the users sign an agreement stating they are ≥18 years old and will adhere to these rules. Diligent compliance of rules and regulations by operators is doubtful as observed by Trivedi et al., which subsequently raises considerable medical and safety concerns regarding their use [5].

The Bird® and Lime ® SESs were introduced in the Indianapolis metropolitan area in June 2018 [6]. Given this recent introduction and axiomatic hazardous use in our Midwest city, we sought to evaluate the pattern of injuries related to the use of SESs.

## Materials and methods

Study design

After obtaining institutional review board approval, the electronic medical records of potential patients of any age presenting to the emergency department (ED) of downtown level I trauma centers in Indianapolis were identified using the search terms “bird”, “lime”, and/or “scooter”. We retrospectively analyzed de-identified data from all records for SES injuries between June 1, 2018, and December 31, 2018. Records were included if the patient injury specifically involved the use of an SES. Records were excluded if the injury description or mechanism did not explicitly identify SES use. Data endpoints collected included: (1) date and time of injury; (2) mechanism of injury; (3) patient use of helmets and alcohol; (4) injury classification and location; (5) diagnostic imaging; and (6) management of patient injuries.

Statistical analysis

Statistical analyses were performed using GraphPad Prism 8.0 (GraphPad Software, San Diego, CA). Continuous and discrete data were reported as mean ± standard deviation. Nominal data were reported as number (percentage). Continuous and discrete variables were analyzed using the two-sided student t-test or two-sided Mann-Whitney U-test when appropriate. Nominal variables were evaluated using Pearson’s χ2 test or two-sided Fisher’s exact test. Odds ratios (OR) and 95% confidence intervals (CI) were calculated for each nominal characteristic. Significance was determined at p < 0.05.

## Results

Demographic characteristics

A total of 89 (n=89) patients were included in our study. A summary of patient demographics is provided in Table 1. Our cohort consisted predominantly of males (n=58, 65.2%) with an average age of 29.2 ± 12.9 years. Six patients (6.7%) were under the legal SES riding age (i.e. ≤18 years).

**Table 1:** Patient and injury characteristics * Sums exceeds patient total (89), as some individuals experienced multiple types of injuries concurrently

Characteristic	Number (%)
Demographics	
Age (years), mean ± SD	29.2 ± 12.9
<18y	6 (6.7)
18y – 29y	46 (51.7)
30y – 44y	26 (29.2)
45y – 64y	7 (7.9)
≥65y	4 (4.5)
Sex (M)	58 (65.2)
Injury Characteristics	
Day	
Monday	14 (15.6)
Tuesday	8 (9.0)
Wednesday	11 (12.4)
Thursday	11 (12.4)
Friday	11 (12.4)
Saturday	22 (24.7)
Sunday	12 (13.5)
Time	
0600 – 1359	11 (12.4)
1400 – 2159	49 (55.1)
2200 – 0559	23 (25.8)
Unknown	6 (6.7)
Mechanism of Injury*	
Fall	41 (46.1)
Collision	17 (19.1)
No Description	37 (41.6)
Alcohol Intoxication	13 (14.6)
Helmet Use	
Wearing	0
Not Wearing	59 (66.3)
Unknown	30 (33.7)
Type of Injury*	
Abrasion/Contusion	29 (32.6)
Fracture	24 (27.0)
Laceration	16 (18.0)
Joint/Ligament Injury	5 (5.6)
Concussion	3 (3.4)
Dental Injury	3 (3.4)
Muscular Strain/Sprain	30 (33.7)
Injury Location*	
Head & Neck	38 (42.7)
Face	30 (33.7)
Scalp/Brain	11 (12.4)
Neck	1 (1.1)
Upper Extremity	32 (36.0)
Lower Extremity	27 (30.3)
Trunk	11 (12.4)

Overall injury characteristics

A summary of injury characteristics is also provided in Table 1. Patients most frequently presented to the ED on Saturday (n=22, 24.7%) from 14h00 to 21h59 (n=49, 55.1%). Patients were injured either due to a fall (n=4, 46.1%) or due to a collision (n=17, 19.1). No clear description of the injury mechanism was provided in 41.6% of records (n=37). Thirteen (14.6%) of the injuries were associated with alcohol intoxication. No individual was recorded to have worn a helmet at the time of injury, although 33.7% of reports (n=30) did not indicate its use or disuse.

The most common types of injury were contusions and/or abrasions, with a total of 43 were noted in 30 patients (33.7%). Of these, 53.3% involved the lower extremities (n=16), 40.0% involved the upper extremities (n=12), and 33.3% involved the head and neck (H&N) (n=10). A total of 32 fractures were identified in 29 patients (32.6%), with 14 fractures (43.8%) involving the upper extremities, six fractures (18.75%) involving the maxillofacial region, six fractures (18.8%) noted in the thorax/abdomen, five fractures (15.6%) involving the lower extremities, and one fracture (3.13%) involving the skull. Lacerations were noted in 24 patients (27.0%). Interestingly, all lacerations occupied the H&N region, with 83.3% specifically involving the facial region. Sixteen (18.0%) incidences of joint or ligament injury were noted, comprised 10 upper extremity injuries (62.5%, seven shoulder, three hand/wrist) and six lower extremity injuries (37.5%, four knee, two foot/ankle). Less frequently diagnosed injuries included concussions (n=5, 5.6%), dental injuries (n=3, 3.4%), muscular strains or sprains (n=3, 3.4%), and intraventricular hemorrhage (n=1, 1.1%). No patient deaths were noted from the use of SESs.

Furthermore, our findings demonstrate that patients were most likely to experience trauma to the H&N region (n=38, 42.7%), with the maxillofacial region being the most commonly affected subsite in the head and neck (n=30, 71.4%) while isolated neck injuries were the least frequent (n=1, 2.4%). Brain/scalp injuries were noted in 11 records (26.2%) while upper extremity and lower extremity injuries were noted in 32 (36.0%) and 27 (30.3%) cases, respectively. Thoracoabdominal injuries were the least common (n=11, 12.4%).

Radiographic imaging

Table 2 summarizes the diagnostic imaging obtained for patients in our cohort. At least one radiographic image of any type was acquired in 79.8% (n=71) of patients. A total of 71 computed tomography (CT) scans and 101 X-ray (XR) image series were obtained, with a mean of 0.7 ± 1.2 CT scans and 0.8 ± 0.7 XR imaging series per case. Two or more diagnostic imaging tests were obtained for 27 records (30.3%). No magnetic resonance (MR) or ultrasound images was utilized.

**Table 2:** Diagnostic imaging workup by injury location Totals may exceed the listed maximum as individuals may have received multiple radiographs of each category

	Imaging Type, No. (%)	
Anatomical Location	CT	XR
Head & Neck, Any*	25 (28.1)	0 (0.0)
Maxillofacial	14 (15.7)	0 (0.0)
Head/Brain	20 (22.5)	0(0.0)
Neck/Spine	15 (16.9)	0 (0.0)
Upper Extremity, Any	1 (1.1)	38 (42.7)
Shoulder Girdle	1 (1.1)	13 (14.6)
Arm	0	5 (5.6)
Elbow	0	10 (11.2)
Forearm	0	6 (6.7)
Hand & Wrist	0	19 (21.3)
Lower Extremity, Any	1 (1.1)	20 (22.5)
Thigh	0	0
Knee	1 (1.1)	13 (14.6)
Leg	0	5 (5.6)
Foot & Ankle	0	14 (15.7)
Chest	5 (5.6)	10 (11.2)
Abdomen/Pelvis	4 (4.5)	5 (5.6)
Thoracic/Lumbar Spine	1 (1.1)	1 (1.1)

At least one H&N CT scan was obtained in 25 (28.1%) patients, many of whom required multiple CT scans, resulting in a total of 49 CT series (total = 49). For the H&N, 20 scans (40.8%) were of the head, 15 scans (30.6%) were of the neck and/or cervical spine, and 14 scans (28.6%) were of the maxillofacial area. A chest or abdominopelvic CT was acquired in five (5.6%) and four (4.5%) patients, respectively. Of note, none of the XR series obtained was of the H&N. The distribution of XR imaging for other regions of the body is summarized in Table 2.

Management and treatment

All lacerations (n=24, 27.0%) were repaired utilizing sutures and/or butterfly stitches with a skin adhesive. Nine individuals (10.1%) with abrasions underwent wound cleansing and application of topical antibiotic ointment. Five patients (5.6%) required bedside reduction of a fracture or dislocation, and 30 patients (34.8%) had conservative orthopedic management with slings, splints, casts, and/or crutches. Pain management was administered as a nonsteroidal anti-inflammatory drug (NSAID) in 30 individuals (34.8%) while opioids were prescribed to only four patients (4.5%).

A total of 12 patients (13.5%) underwent an operative procedure under general anesthesia. Of these, five patients (5.6%) received open reduction and internal fixation for fractures while three patients (3.4%) were fixated externally. One individual required repair for complete acromioclavicular ligament separation. Dental injuries (n=3, 3.4%) were managed with oral surgery after discharge from the ED. Five (5.6%) patients were admitted to the hospital for >24 hours, all of whom required surgery.

Subset analysis of head & neck injuries

A comparison of factors associated with H&N injuries (HNIs) is found in Table 3. Patients with HNIs were older as compared to patients without HNIs (33.8 ± 16.1 vs. 25.7 ± 8.4 years, p = 0.003), but no difference was noted between sex distribution (p = 0.180), day of the week (p = 0.506), or time of day (p = 0.224). However, individuals that sustained HNI were more frequently intoxicated with alcohol (29.0% vs. 3.9%, p = 0.002, OR = 9.98). All laceration injuries (n=24) involved the H&N (p <0.001). No statistical difference was observed comparing abrasion/contusion rates (p = 0.629) or rates of any fracture occurrence (p = 0.067).

**Table 3:** Factors associated with head & neck injuries Comparison of the patient factors associated with head and neck (H&N) injuries versus non-H&N injuries. Continuous variables are represented as mean ± SD, and categorical variables are represented as number (percent). a 2-sided, unpaired Student’s t-test b 2-sided, unpaired Fisher’s exact test c 2-sided, unpaired Pearson’s χ2 test d 2-sided, unpaired Mann-Whitney U-test

Characteristic	H&N Injury (n=38)	Other Injury (n=51)	p-value	OR (95% CI)
Age	33.8 ± 16.1	25.7 ± 8.4	0.003^a^	
Sex (M)	28 (73.7)	30 (58.8)	0.180^b^	1.96 (0.77 – 5.15)
Day of Week			0.506^b^	1.46 (0.60 – 3.58)
Sun – Thurs	22 (57.9)	34 (66.7)		
Fri – Sat	16 (42.1)	17 (33.3)		
Time of Day			0.224^c^	
0600 – 1359	4 (10.5)	7 (13.7)		
1400 – 2159	20 (52.6)	29 (56.9)		
2200 – 0559	14 (36.8)	9 (17.6)		
Alcohol Intoxication	11 (29.0)	2 (3.9)	0.002^b^	9.98 (2.04 – 46.61)
Injury				
Laceration	24 (63.6)	0	<0.001^b^	∞
Abrasion/Contusion	12 (42.9)	18 (35.3)	0.629^b^	1.38 (0.53 – 3.28)
Fracture	8 (21.1)	21 (41.2)	0.067^b^	0.38 (0.14 – 1.02)
Imaging				
Any CT	23 (60.5)	5 (9.8)	<0.001^b^	14.11 (4.61 – 37.77)
Any XR	15 (39.5)	45 (88.2)	<0.001^b^	0.09 (0.03 – 0.25)
Total CT	1.4 ± 1.6	0.1 ± 0.4	<0.001^d^	
Total XR	0.6 ± 0.9	1.0 ± 0.5	0.001^d^	
Treatment				
Operative	4 (10.5)	8 (15.7)	0.546^b^	0.63 (0.20 – 2.07)
Laceration Repair	24 (63.6)	0	<0.001^b^	∞
Pain Management	9 (23.7)	21 (41.2)	0.113^b^	0.44 (0.19 – 1.15)
Wound Management	5 (13.2)	4 (7.8)	0.488^b^	1.78 (0.48 – 6.12)

When compared to the non-HNI group, the HNI group had a statistically higher rate of CT imaging (60.5% vs. 9.8%, p <0.001, OR = 14.11) but had lower rates of XR imaging (39.5% vs. 88.2%, p <0.001, OR 0.09). Overall, the HNI group had lower rates of radiographic imaging (68.4% vs. 88.2%, p = 0.032, OR = 0.29). No difference was observed between the frequencies of operative management (10.5% vs. 15.7%, p = 0.546, OR = 0.63), bedside wound care (13.2% vs. 7.8%, p = 0.488, OR = 1.78), or isolated analgesic therapy (23.7% vs 41.2%, p = 0.113, OR = 0.44) in the HNI subgroup.

## Discussion

The idea of transportation-sharing began in Amsterdam, the Netherlands, in 1965, with the free, public “White Bicycle Plan” [7]. Developed by the Dutch anarchist group “Provo” to reduce pollution, cost, and congestion, the plan quickly failed as the bicycles were vandalized, destroyed, or stolen [8]. Several similar attempts have failed over the decades until the 2000s when trackable technology and electronic payments were applied to bike-sharing systems. This idea gained exponential popularity and was adopted to other forms of transport-sharing systems, including SESs, in 2017 [1,8].

Since their introduction to American public transportation in Southern California, SESs have increasingly gained public approval [5,9-11]. This is largely due to the eco-friendly, convenient, and affordable nature of SESs. Additionally, the ubiquitous availability of SESs in urban settings coupled with the ability to arbitrarily “park” SESs on sidewalks has catalyzed their acceptance in the public’s view.

However, with this increased availability and use, the presumed public safety hazard has gained substantial evidence due to nonadherence to traffic laws, helmet usage, age restrictions, and operation while intoxicated [5,9-11]. The authors predict that as the popularity of SESs continues to rise, safety issues and injuries will also increase proportionally.

The results of our investigation validate those of recent SES trauma reports. The demographics have consistently shown a 50% - 60% male predominance, with an average age of early- to mid-30s [5,9-11]. Racial distribution has only been analyzed by Aizpuru et al., who reported a slight white predominance (54.8%) across a nationwide database system [10]. Injuries have more frequently occurred during the afternoon and evening hours on weekends when SESs see the most use [5,9]. Although studies have reported rates of helmet use ranging between 0% and 6.3%, the true rate remains elusive, as all studies have been retrospective and have admitted varying degrees of incomplete documentation [5,9-11]. Passive, subjective observation of active SES riders by the authors in our city, as well as other urban settings, supports these findings [5]. Although Trivedi et al. reported a 4.8% prevalence rate of alcohol intoxication, the findings from our cohort (14.6%) better align with the intoxication rate of 16% published by Badeau et al. (16%) [5,11].

Certain striking similarities have emerged in the trends of injury mechanisms and injury types as demonstrated by several reports investigating SESs injuries. Superficial soft tissue injuries (abrasions, contusions, and lacerations) and orthopedic injuries (fractures, dislocations, and strains/sprains) tend to dominate the injury type profile [5,9-11]. Falls are the most common mechanism of injury followed by collisions while severe life-threatening/life-ending injuries remain rare. Furthermore, although most injuries are managed on an outpatient basis, injuries requiring inpatient admission or surgical management are not uncommon, especially for major orthopedic injuries [5,9-11].

HNIs feature prominently in all SES trauma studies [5,9-11]. Although categorizations vary, rates of HNI range from 27.6% to 44.4% of patients [5,9-10]. Many of the injuries are superficial in nature, but more serious afflictions, such as concussions, intracranial hemorrhages, and fractures, are frequent enough to warrant both medical and legal concerns [5,9-11]. What remains unknown are the effects helmet use would have on these HNIs. As evidenced by bicycle helmet use, operation with helmets would significantly reduce the overall frequency and intensity of head and maxillofacial injuries, though lower face injuries would be protected to a lesser degree [12-13].

Interestingly, our findings are strikingly similar to injuries related to non-motorized stand-up scooters. As reported by Mebert et al., with a 44.2% prevalence rate of fractures, of which 28% involved the craniofacial region. Injuries were also noted to predominantly involve young males without safety helmets [14].

Other self-propelled transportation devices have demonstrated similar injury profiles to SESs. Hoverboards exhibit a nearly identical fracture and soft tissue injury profile, with the exception of a higher pediatric prevalence rate of 47% to 61% [15-16]. Although HNIs are less frequent with Segway® (Bedford, New Hampshire) personal transporters (15.6%), which tend to occur in older individuals with a mean age of 48 years, soft tissue injuries, and orthopedic injuries remain the most common injury types [17]. While bicycle accidents favor slightly older males and produce more frequent and severe intracranial damage, skateboard and longboard injuries affect younger males (88%) with similar fracture and soft tissue damage rates, predominantly occurring in the extremities [18-19].

The financial burden of SES injuries has yet to be studied [5,9-11]. Despite lacking precise data, we can infer that SES injuries substantially add to healthcare expenditure. Most individuals presenting to the ED require some form of imaging, often in the form of CT scanning. In addition to the direct costs, diagnostic imaging tests increase radiation exposure.

A possible avenue that can be utilized to mitigate SES-related injuries is the requirement for helmet use and the imposition of legal consequence for a lack thereof. The implementation of mandatory bicycle helmet laws has been successful at reducing rates of head injury [20]. Infrastructure modifications, such as bicycle lanes, have also contributed to safety improvements [21-22]. Plans for helmet-sharing programs have been discussed but logistical and hygienic issues have prevented large-scale implementation [23]. Furthermore, extending legal consequences for reckless SES operation may also help with reducing incidence rates of SES-related injuries.

Design alterations may contribute to improving the safety profile of SESs. Currently, SESs have a narrow, hard wheel designed primarily for use on smooth surfaces. However, in real-world conditions, users often encounter uneven surfaces. The replacement of narrow, hard wheels with wider, softer wheels would provide additional traction to neutralize the destabilizing effect of uneven surface topography. Wider platforms for the feet may provide greater stability and reduce slipping. Additionally, regular maintenance of brakes, lights, and tires are necessary to ensure optimal safety and performance.

To the best of our knowledge, this is the first study to analyze the use of SESs specifically in a Midwest urban setting. Moreover, it is the first to analyze the factors associated with HNI related to the use of SESs. However, there are several limitations to our study. It is retrospective in nature with limited accessibility to certain data endpoints. Our information was collected from EDs serving a single urban population and, therefore, subject to sampling bias. Visits to primary care offices, specialists, and urgent care for SES-related injuries were not obtained. The small sample size may not be truly representative of the Midwestern population, and the limited time frame of data collection may skew data as our region experiences substantial seasonal weather changes. Future prospective trials are required to reduce data gaps, particularly with the mechanism of trauma, helmet use, and the financial burden of these injuries.

## Conclusions

The SES is a simple, widely available, and ecofriendly transportation modality that has rapidly gained widespread popularity. However, nonadherence to safety guidelines and reckless operation can result in significant injuries and medical costs. Further data acquisition and evidence are needed to implement safer SES policies.

## References

[1] Hall M. (2019). Bird scooters flying around town. Santa Monica Daily Press..

[2] (2019). Lime electric scooter rentals. https://www.li.me/electric-scooter.

[3] (2019). Bird company. https://www.bird.co/.

[4] (2019). SPIN company. https://www.spin.app/.

[5] Trivedi TK, Liu C, Antonio ALM (2019). Injuries associated with standing electric scooter use. JAMA Netw Open.

[6] (2019). Another electric scooter service arrives in Indianapolis. https://www.indystar.com/story/news/2018/06/22/lime-dockless-scooter-service-coming-indianapolis/725641002/.

[7] (2019). Story of cities #30: how this Amsterdam inventor gave bike-sharing to the world. https://www.theguardian.com/cities/2016/apr/26/story-cities-amsterdam-bike-share-scheme.

[8] Schroeder B, Hagen J, Leve Z, Peñalosa A (2019). Bike-sharing goes viral. June 10, 2019.

[9] Mayhew LJ, Bergin C (2019). Impact of e-scooter injuries on emergency department imaging. J Med Imaging Radiat Oncol.

[10] Aizpuru M, Farley KX, Rojas JC, Crawford RS, Moore TJ, Wagner ER (2019). Motorized scooter injuries in the era of scooter-shares: a review of the national electronic surveillance system. Am J Emerg Med.

[11] Badeau A, Carman C, Newman M, Steenbilk J, Carlson M, Madsen T (2019). Emergency department visits for electric scooter-related injuries after introduction of an urban rental program. Am J Emerg Med.

[12] Benjamin T, Hills NK, Knott PD, Murr AH, Seth R (2019). Association between conventional bicycle helmet use and facial injuries after bicycle crashes. JAMA Otolaryngol Head Neck Surg.

[13] Høye A (2018). Bicycle helmets - to wear or not to wear? A meta-analyses of the effects of bicycle helmets on injuries. Accid Anal Prev.

[14] Mebert RV, Klukowska-Roetzler J, Ziegenhorn S, Exadaktylos AK (2018). Push scooter-related injuries in adults: an underestimated threat? Two decades analysed by an emergency department in the capital of Switzerland. BMJ Open Sport Exerc Med.

[15] McIlvain C, Hadiza G, Tzavaras TJ, Weingart GS (2019). Injuries associated with hoverboard use: A review of the National Electronic Injury Surveillance System. Am J Emerg Med.

[16] Weingart GS, Glueckert L, Cachaper GA, Zimbro KS, Maduro RS, Conselman F (2017). Injuries associated with hoverboard use: a case series of emergency department patients. West J Emerg Med.

[17] Pourmand A, Liao J, Pines JM, Mazer-Amirshahi M (2018). Segway® Personal Transporter-related injuries: a systematic review and implications for acute and emergency care. J Emerg Med.

[18] de Guerre LEVM, Sadiqi S, Leenen LPH, Oner CF, van Gaalen SM (2018). Injuries related to bicycle accidents: an epidemiological study in The Netherlands. Eur J Trauma Emerg Surg.

[19] Keays G, Dumas A (2014). Longboard and skateboard injuries. Injury.

[20] Høye A (2018). Recommend or mandate? A systematic review and meta-analysis of the effects of mandatory bicycle helmet legislation. Accid Anal Prev.

[21] Gu J, Mohit B, Muennig PA (2017). The cost-effectiveness of bike lanes in New York City. Inj Prev.

[22] Smith A, Zucker S, Lladó-Farrulla M (2019). Bicycle lanes: are we running in circles or cycling in the right direction?. J Trauma Acute Care Surg.

[23] (2019). You share scooter, what about helmets? Startup works on environmentally friendly helmet sharing program to complement micro-mobility vehicle sharing programs. https://thepacificsentinel.com/share-scooters-helmets/.

